# A qualitative evidence synthesis of employees’ views of workplace smoking reduction or cessation interventions

**DOI:** 10.1186/1471-2458-13-1095

**Published:** 2013-11-26

**Authors:** Christopher Carroll, Jo Rick, Joanna Leaviss, David Fishwick, Andrew Booth

**Affiliations:** 1 Health Economics and Decision Science (HEDS), School of Health and Related Research (ScHARR), University of Sheffield Regent Court, Regent Street, Sheffield S1 4DA, UK; 2 Institute of Population Health, Manchester Academic Health Science Centre, University of Manchester, Williamson Building, Manchester M13 9PL, UK; 3 Centre for Workplace Health, Health & Safety Laboratory, Sheffield Teaching Hospitals NHS Foundation Trust and the University of Sheffield, Sheffield, UK

## Abstract

**Background:**

The need to reduce smoking rates is a recognised public health policy issue in many countries. The workplace offers a potential context for offering smokers’ programmes and interventions to assist smoking cessation or reduction. A qualitative evidence synthesis of employees’ views about such programmes might explain why some interventions appear effective and others not, and can be used to develop evidence-based interventions for this population and setting.

**Methods:**

A qualitative evidence synthesis of primary research exploring employees’ views about workplace interventions to encourage smoking cessation, including both voluntary programmes and passive interventions, such as restrictions or bans. The method used was theory-based “best fit” framework synthesis.

**Results:**

Five relevant theories on workplace smoking cessation were identified and used as the basis for an *a priori* framework. A comprehensive literature search, including interrogation of eight databases, retrieved 747 unique citations for the review. Fifteen primary research studies of qualitative evidence were found to satisfy the inclusion criteria. The synthesis produced an evidence-based conceptual model explaining employees’ experiences of, and preferences regarding, workplace smoking interventions.

**Conclusion:**

The synthesis suggests that workplace interventions should employ a range of different elements if they are to prove effective in reducing smoking among employees. This is because an employee who feels ready and able to change their behaviour has different needs and preferences from an employee who is not at that stage. Only a multi-faceted intervention can satisfy the requirements of all employees.

## Background

The need to reduce smoking has become a major policy and public health issue in recent years, due to the well-documented burden of smoking-related diseases on individual health and society as a whole. The workplace is seen as an appropriate context for assisting smoking cessation because it enables access to a large and fairly stable population, facilitating higher participation rates for interventions, and with the potential to use peer group support and positive peer pressure [1]. The United Kingdom has introduced national workplace, smoke-free policies in the last 10 years and the National Institute of Health and Care Excellence has produced guidelines on how to implement these policies and how best to assist employees with smoking cessation [2]. Previous systematic reviews have found that smoke-free workplaces encourage quitting and reduction in smoking rates [3] and that proven stop-smoking methods (i.e. from outside the workplace), including group therapy, individual counselling and nicotine replacement therapy, are equally effective when offered in the workplace [1]. Such interventions have also been found to be cost-effective [4]. The evidence has been found to be more equivocal for other approaches, such as self-help methods, social and environmental support, and incentives and competitions [1].

There is currently no synthesis of qualitative evidence related to workplace smoking cessation programmes, but the value of such a synthesis is twofold. First, a consideration of the qualitative evidence of employees’ views regarding workplace interventions or programmes might help to explain outcomes, that is, why some interventions might work and others not. This is one function of qualitative evidence synthesis [5,6]. Second, many of the non-pharmaceutical interventions for smoking cessation or reduction are so-called “complex interventions”, that is, “interventions with several interacting components” [7]. It is recommended that complex interventions be developed based on theory [7], but qualitative evidence synthesis offers an alternative approach from theory for identifying appropriate and acceptable elements of such interventions [8].

### Objectives

The objective of the present work therefore was to perform a qualitative evidence synthesis of employees’ views and experiences of workplace smoking cessation strategies or interventions. This was undertaken in order to understand why certain interventions might not work, or appear not to work, and to identify potentially relevant components for an intervention in this field.

## Methods

There are many recognised theories that seek to explain health behaviours, such as the Transtheoretical model of behaviour change [9], so it makes sense to utilise such models and theories in evidence syntheses that address questions relating to health behaviour or decision-making [10]. Synthesis methods that adopt this approach include realist and framework synthesis [11-13] and “best fit” framework synthesis [8,14]. The “best-fit” method was chosen as it has been found by the authors to be suitable for questions relating to individuals’ decision-making regarding health behaviours [8,14]. It explores relevant theory within a specific context, and can generate a refined, context-specific, conceptual model that can be used to understand the reported effectiveness or otherwise of interventions, as well as being used itself to develop interventions. The method is described in full elsewhere [8,14]. In essence, it involves the identification of relevant theories or conceptual models to create an *a priori* framework. Data from primary research studies identified for the synthesis are coded against this *a priori* framework; data not captured by this framework are then analysed using secondary thematic analysis to generate new themes. A new framework and conceptual model is thus created using all themes, pre-existing and newly specified, found by reviewers within the data.

### The a priori framework

In this synthesis the framework was based on three theories relating specifically to smoking cessation or reduction behaviours: The Transtheoretical Model (TTM) of Behaviour Change, including its related Stages and Processes of Change elements [9], the Theory of Planned Behaviour (TPB) [15] and the Health Belief Model (HBM) [16]. These theories had been adapted specifically for use in workplace intervention studies in five identified articles: Three papers reported conceptual models based on the TTM [17-19], one on the TPB [20] and one the HBM [21]. The various components of these theories and models were used to develop the *a priori* framework for analysis, which is given in Table 1. The methods used for identifying the theories and creating the *a priori* framework are described in detail elsewhere [8].

**Table 1:** The coding framework

**Concepts derived for coding**	**Definitions**
Beliefs about smoking	Person considers there to be or not to be a problem
Perceived pros and cons of smoking	Person beginning to consider benefits of change; Perceived susceptibility to disease (I don’t think anything will happen to me *vs* my family has a history)
	Perceived seriousness of disease (not bothered *vs* very concerned)
Perceived norms regarding smoking	A person accepting or participating, or rejecting or not participating, in the programme because it is expected of them
Priority of quitting	It is/is not important to me; I see it as urgent, to be done soon *vs* no rush
Perceived ability to quit	A person’s confidence in their ability to take action and persist in action: I feel able or unable to quit; or I feel the programme provides me with the motivation to quit; self-efficacy
Dependence	A person considers themselves to be addicted and nothing will work; or no programme works; they have tried quitting before but without success
Social support	It was very helpful to have the support of my: Friends; Family
Organisation support	The work environment is/is not conducive to quitting smoking
Opportunity	A person participates because the programme is available
Substitutes	Substitution of alternatives to the problem behaviour
Incentives to quit	Receiving a reward for making the change. The provision of items such as money, prizes and products, or some form of self-reward, which are intended to motivate smokers to reduce consumption or quit

### Searching and study selection

An evaluated search strategy was developed for identifying primary qualitative research studies for inclusion in the qualitative review [22]. This was run in December 2011 and involved combining free text and database thesaurus terms for workplace or employees with terms for smoking cessation or health promotion, and terms for qualitative research. See Additional file 1. The following databases were interrogated to identify relevant published and unpublished literature: Social Science Citation Index, PsycINFO, CINAHL, ASSIA, IBSS, Emerald reviews, ERIC and MEDLINE. The reference lists of all papers included in the review were also checked for additional relevant citations. Two reviewers (JR, JL) conducted independent screening of all citations. Discrepancies were resolved by discussion. Full papers of all potentially relevant citations were retrieved and inclusion criteria applied. Any disagreements over inclusion were resolved with reference to a third reviewer.

### Inclusion criteria

The synthesis included all types of studies reporting employee’s views about workplace smoking cessation strategies or interventions, including interviews, focus groups and satisfaction surveys, which might use open questions or quantify people’s views or preferences in terms of frequencies. Only studies from north America, Australasia and western and northern Europe were to be included, as these regions were considered to be sufficiently similar both economically and in terms of the behaviour of interest [23], and thus to accommodate the combining of studies in a synthesis.

### Data extraction and synthesis

A data extraction form was developed based on the key data required for the synthesis, including details of the population, setting and intervention. It also included the concepts of the *a priori* framework. Data for analysis were extracted from the Results sections of papers and consisted either of verbatim quotations from study participants or findings reported by authors. Three reviewers independently piloted the form on two studies, before finalising an agreed version of the form. For all papers, two reviewers (JL, JR) each independently extracted data and coded Results data against the *a priori* framework. This coding was then supplemented by secondary thematic analysis of any data not captured by the framework. The method applied is described in detail elsewhere [8,14].

### Quality assessment, sensitivity and dissonance

The two reviewers (JR, JL) conducted independent quality assessment of the included studies using published criteria based on the quality of a study’s reporting across four domains: A study’s design, and methods of sampling, data collection and analysis [24]. These assessments were used to inform judgments on both the internal validity of the studies and, consequently, the validity of the synthesis findings. The latter was tested by a post-synthesis assessment of “dissonance” [8,25] and the effect of excluding “inadequately-reported” studies from the synthesis.

## Results

**Figure 1:**
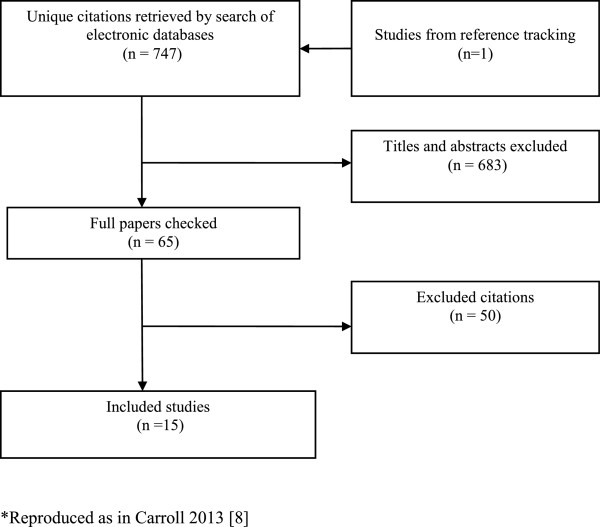
PRISMA flowchart detailing search and study selection process.

The search generated 747 unique citations. Sixty-five full papers were retrieved as potentially relevant, of which 14 studies were found to satisfy the inclusion criteria. One additional relevant study was identified from the references of an included study [26]. See Figure 1.

For the characteristics of the included studies, see Table 2. Six studies examined people’s views about employers’ decisions to restrict smoking within or at a workplace. Five studies explored views relating to multi-faceted interventions, i.e. involving a combination of at least two or more of the following: self-help or educational materials, smoking cessation resources or “props” such as nicotine patches, nicotine replacement therapy (NRT) or pencil cigarettes, support groups, peer support, telephone counselling, and competitions or incentives. One employed telephone counselling only and one incentives only. Two studies did not specify the intervention, but rather elicited people’s views on the principle of a workplace smoking cessation intervention. Where reported, there was considerable variety in terms of organisation sector and size. Eight studies employed satisfaction surveys with closed questions, and seven studies employed one-to-one interviews, focus groups, discussion group posts or open-ended survey questions.

**Table 2:** Study characteristics

**Study**	**Location**	**Design**	**Objective**	**Sector(s)**	**Sample (N)**	**Male (%)**	**Other socio-demographic data**	**Nature of smoking cessation programme**	Quality assessment*
Bondy 2011 [27]	Canada	Qualitative: An analysis of discussion board posts	To understand social context and smoking restrictions and identify barriers and facilitators to achieving smoke-free workplaces	Construction	250	89	NR	Restrictions on smoking in the workplace	Adequate
Borland 1997 [28]	Australia	Quantitative: Questionnaire	To understand employees’ beliefs and opinions about their own smoking and the smoking ban	Medium-sized workplaces	794	50	“High blue collar percentage”	Restrictions on smoking in the workplace	Adequate
Croucher 2007 [29]	UK	Qualitative: Focus groups and individual semi-structured interviews	To understand the issues of creating smoke-free work environment, and controlling the availability of alternatives	Catering	81	100	Aged 18–65, Bangladeshi	Proposed restrictions on smoking in the workplace	Adequate
Eadie 2010 [30]	UK	Qualitative: Individual in-depth interviews	To understand the impact of smoking legislation within the bar community	Service/Hospitality	26	38	Aged 30-49 = 69%	Restrictions on smoking in the workplace	Adequate
Fisher 1994 [31]	USA	Quantitative: Survey	To understand employees’ perceptions of social support	Clerical, ManufacturingProfessional services	98	NR	NR	Self-help materials, support groups	Inadequate
Glasgow 1991 [32]	USA	Quantitative	To assess attitudes and beliefs about smoking, awareness of smoking control activities, and participation in these activities	Wholesale, Service, Manufacturing	Unclear	50	NR	Presentations, workshops, contests/competitions; self-help materials; worksite networks	Adequate
Harley 2010 [33]	USA	Qualitative: Discussion groups facilitated by a semi-structured topic guide	To understand employees’ experiences of smoking and healthy eating	Construction/Labouring	300	90	“Blue collar workers”; majority white	Telephone counselling, some support groups	Adequate
Hunt 2007 [34]	USA	Quantitative: Survey	To identify factors predictive of teen smoking and to record participants’ experiences of an intervention	Retail	252	52	Majority (64%) white	Contests, games, demonstrations, peer leaders and advisory boards at work; incentives; educational materials; materials in break rooms	Adequate
Janke 2010 [26]	USA	Qualitative: In-depth interviews	To understand the current climate of tobacco control in the military	Military	52	17	NR	Restrictions and bans	Adequate
Kim 2011 [35]	USA	Qualitative: A series of open-ended questions by telephone interview	To understand how and why incentives were or were not effective	NR: “A multinational”	36	NR	NR	Financial incentives	Adequate
Olsen 1991 [36]	USA	Quantitative: Survey	To examine recidivism among 6-month quitters	Chemical industry	Subset of 1258	90	NR for the subset	Buddy program, self-help materials, group clinics, nicotine gum, incentive prizes	Adequate
Osuchow-ski 2009 [37]	Poland	Quantitative: Survey	To understand employees’ perceptions of risk from smoking, their expectations of employer, and willingness to join programmes	“A large plant”	1412	NR	NR	Unspecified: The principle of a workplace smoking cessation programme	Inadequate Abstract only
Powell 1993 [38]	USA	Quantitative: Survey	Unclear	Manufacturing	622	NR	25% blue collar; 75% white collar	Guided self-help materials, telephone counselling, cigarette “props”, eg. cigarette pencil, “urge zapper”, etc.	Inadequate
Styles 1998 [39]	UK	Quantitative: Questionnaire	To understand smoking behaviour and cessation intentions of smokers	Retail, Service, Heavy Industry, white collar	242	59	Age range: 17–64 years	Restrictions on smoking in the workplace	Adequate
Tiede 2007 [40]	USA	Qualitative: Focus groups with detailed question guide	To understand perceived workplace pressures to quit and attitudes towards existing cessation resources and initiatives	Manufacturing/Labour, Service/Hospitality	59	31	NR	Unspecified: The principle of a workplace smoking cessation programme	Adequate

NR: Not reported.

*If a paper had clearly reported information on how the study was conducted relating to two or more of the four criteria, then it was categorised as “Adequate”; if it reported information on study conduct relating to only one or none of the quality assessment criteria, then it has been categorised as “Inadequate”. The two reviewers were completely consistent in their independent categorisation of the studies by quality.

### Synthesis and the conceptual framework

Data from the included studies were found to support all concepts from the *a priori* framework, i.e. none was dropped from the final synthesis because of an absence of evidence in the included studies. Reviewers generated six new concepts from interpretation of the data, all of which related to the context for the behaviour (smoking), i.e. either the setting, including roles and responsibilities of the employer in this area (employer obligations, employer responsibilities, and enforcement), or the interventions themselves (ease and convenience; alternatives and cost; and co-worker interaction). For the new concepts, see Table 3.

**Table 3:** New concepts from the secondary thematic analysis

**New concepts**	**Definitions**
**Employees’ expectations of employers**	
Obligations	The necessity for employers to comply with formal regulations regarding the law on smoking bans or restrictions
Responsibilities	The non-legal responsibilities of employers regarding smoking restrictions or cessation. These might concern either protection for non-smokers or help for smokers
Enforcement	Employees’ experience regarding whether or not legal or other regulations are actually enforced
**Intervention preferences**	
Ease and convenience	The accessibility both of the self-help materials and other types of support, such as counselling or groups
Alternatives and cost	The provision of, and problems associated with such alternatives, such as cost
Co-worker interaction	The use of co-workers within the intervention, such as peer support, support groups, and the institutional encouragement of interventions creating a shared experience. However, co-worker interaction can be negative as well as positive

### Quality assessment and dissonance

The two reviewers independently and consistently categorised each included study in the same way using the criteria outlined above. Only three studies were assessed as “Inadequately reported” [31,37,38] (see Table 2). Following the principles outlined elsewhere [24], the contribution to the synthesis of these three “Inadequately reported” studies was assessed as being limited. Exclusion of these three studies would not have affected either the presence or the detail of any of the concepts in the synthesis. Only one inadequately-reported study [38] contributed a unique insight: The view of participants that the usability of self-help materials might help smokers to engage and be successful with an intervention, an idea not reported elsewhere within included studies. It is therefore likely that the exclusion of these inadequately-reported studies would not have adversely affected the synthesis. Formal procedures to seek possible disconfirming cases were deemed unnecessary because of the ready identification of multiple cases of dissonance, i.e. the presentation of contradictory views. For instance, co-workers and family were reported as acting both positively, as a source of support and shared experience when employees tried to quit smoking, and negatively, as the continued smoking of co-workers and family could also act a barrier to someone being able to quit. The frequent presence of such dissonance, both within individual studies and the evidence as a whole, also reflected the high overall quality of the included studies, and the appropriateness of the evidence for framework and thematic approaches to synthesis.

### Findings

Concepts were clustered and subsumed as “internal attributes” under five higher, abstract concepts, a process described by Morse [41]. In other words, where concepts shared commonalities, those characteristics were reduced to one of five higher concepts relating to the behaviour of interest: *Attitudes to health and the workplace; Readiness for change; Employees’ expectations of their employer*; *Social context* and *Intervention preferences*.

### Employees’ attitudes to health and the workplace

Participants in six studies commented on the *pros and cons of smoking*. This was linked to *beliefs about smoking*. Smokers described the health and social benefits of smoking: Enjoyment [29,35,36], stress reduction or relaxation [35,36], contact with friends and co-workers [29] and concerns about weight gain were they to stop [33,36]. Many non-smokers reported disliking smoking; but there were also smokers and non-smokers who felt there was no problem with smoking either at work or elsewhere [30,37].

Reviewers perceived two distinct findings from studies that explored employees’ experiences of workplace bans or restrictions. First, some smokers and non-smokers thought that smoking was no worse than many other hazards to which people were exposed at work and elsewhere [27,30]. Second, and related, safety in the workplace was an issue described both by smokers and non-smokers, which might be compromised by smoking [27].

Findings in six of the 15 included studies were interpreted under the concept of *perceived norms regarding smoking in the workplace*. This topic often appeared to generate strong feelings from both non-smoking and smoking employees and was found by reviewers almost exclusively in studies that explored workplace bans or restrictions. Perceived norms included employees’ beliefs about their “rights”: The right to smoke, in the face of bans or restrictions, versus the right not to be exposed to others’ smoke in the workplace [26,27,39]. As a result of such different norms, smoking and non-smoking groups could be created within a workplace, with separate identities and aspects of community [26,27,30,39]. This issue was also raised by participants in a series of interviews exploring a hypothetical workplace intervention [40]. Non-smokers tended to approve of smoking restrictions [30] but smokers also reported approving of smoking restrictions, after an initial period of doubt or resistance, when they experienced certain benefits of the ban, e.g. reducing the amount they smoked [27,30].

### Employees’ expectations of their employer

Participants in two studies recognised the issue of *employers’ obligations* regarding the law on smoking bans or restrictions [27,30]. For others, an employer was considered to have a *responsibility* regarding smoking restrictions or cessation, either to protect non-smokers [27,37] or to help smokers to quit [37,40]. The opposite view was also reported from an analysis of survey responses to the idea of an hypothetical workplace intervention: “Only a few stated that their smoking was none of their employer’s business” [40]. Cynicism about health insurance payments and links to tobacco companies coloured some employees’ views about the seriousness with which certain employers took their responsibilities [27,40].

*Employer support* was a related concept. Eight studies using employee interviews, focus groups, discussion board posts or surveys reported that participants found it helpful when their employer was clearly supportive of either smoking restrictions or interventions [26,29,31,34]. Employers could also be supportive by simply making participatory interventions available, thus offering their employees an *opportunity* to access a programme [26,35,38,39]. The issue could be different when the intervention was a workplace restriction or ban. Even if an employer nominally engaged with such an intervention, there might still be an issue with *enforcement*. It was noted by respondents in three studies that a policy restricting smoking might not actually be applied, or that employees themselves might ignore a smoking restriction [26,27,29].

### Intervention preferences

The *ease and convenience* of the intervention was also considered by employees to be important. This might relate to the nature of the materials: “many participants attributed their success to the easy-to-follow, step-by-step program approach of the booklets and cassette tapes” [38]. Alternatively, the failure to make certain resources, such as a counsellor or support groups, available at a convenient time and place, was cited as a barrier to effective participation [26,35]. The *cost* of *alternatives* was also found to be an issue: Some employees felt unable to participate without the provision of free products [26,29,40]. Other issues included replacing one problem with another, for example chewing nicotine gum [29].

Participants held clear views about the potential value of *incentives*. In one interview study and one survey study, participants reported viewing the possibility of prizes or awards as a source of motivation [34,40]: Almost two thirds of participants in one survey study ranked money and prizes as the two greatest motivators for attending smoking cessation activities [34]. Incentives could be financial [35,40] or non-financial [29,34].

The social context of the workplace could also be a factor affecting the potential impact of a programme. *Co-worker interaction* was cited by respondents in a focus group study and a survey study as a factor that might help them to quit, when colleagues were also trying to give-up [31,40], as they represented a source of ideas, support and shared experience. However, the impact of co-workers might also be negative. Survey respondents in one study reported that if co-workers continued to smoke, then this made quitting more difficult [36].

### Readiness for change

Participants in both qualitative and quantitative studies reported being either motivated to quit smoking, and for this reason engaged or keen to engage with the interventions or programmes on offer [35,37,39], or they did not view it as a priority and so there was no such interest [29,37]. Wanting to quit was also strongly related to intervention preferences. As a motivating factor, it “trumped” any incentives on offer, which were deemed to be a happy bonus only, while the simple availability of the programme presented an *opportunity* to be taken only by those employees for whom it was a priority to quit [35]. Several other issues beyond the workplace context also influenced employees’ views. First, there was the problem of addiction. Employed smokers in two studies stated that they recognised that they were dependent, and that no programme or incentive would be sufficient to affect a change in their behaviour [28,35]. This *perceived ability to quit* was particularly apparent as an issue in the studies of smoking bans and restrictions: Borland [28], Eadie [30] and Styles [39] all reported that smokers thought they would find dealing with the regulations, and quitting, extremely difficult. The compulsory nature of the intervention was a source of anxiety for those who were not ready to quit [30].

### Social context

This could also be a factor affecting the potential impact of a programme. While *co-worker interaction* in the workplace might facilitate or hamper quitting, the impact of friends and family outside of the workplace might be equally negative. One survey study reported that participants said that if a spouse continued to smoke, then this made quitting more difficult [36], while another reported that, among the teenagers in their study, it was the attitude and behaviours of friends rather than co-workers or a smoking cessation programme, which was most likely to influence their own behaviour [34].

### From the new conceptual framework to a model and theory

**Figure 2:**
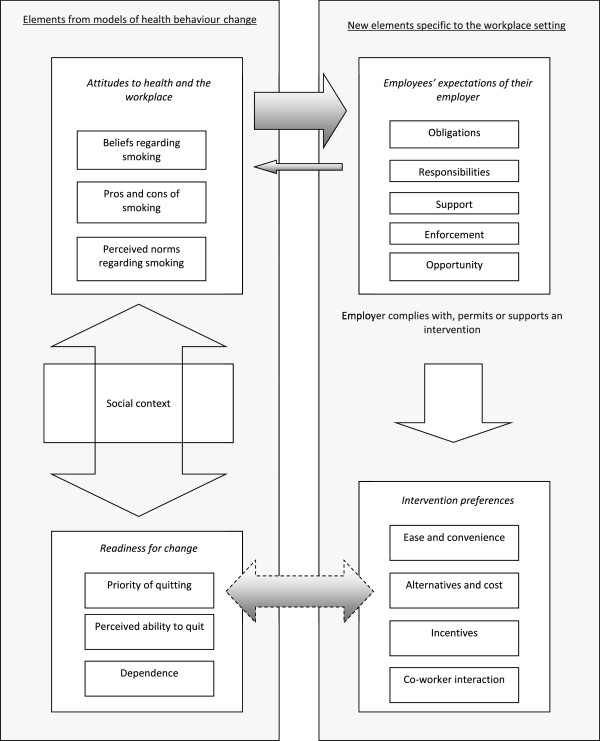
Conceptual model of employees’ views regarding workplace smoking programmes.

The conceptual model resulting from the synthesis is depicted in Figure 2.

The transition from the list of concepts to the model relied on two stages of further reduction and interpretation of the data. First, concepts were clustered and subsumed as “internal attributes” within more abstract concepts [41]. In other words, where concepts shared commonalities, reviewer interpretation of the data reduced those characteristics to, in this case, five higher concepts relating to the behaviour of interest. For example, incentives, ease and convenience, alternatives and cost were all clustered under the higher concept of *Intervention preferences*. Second, these higher concepts and their “internal attributes” were each contextualised with reference to the data to understand the relationships between concepts [41]. For example, an individual smoker’s *readiness for change* was strongly related to views about intervention *preferences*. This is represented by the bi-directional arrow between these concepts (see Figure 2). If employees were provided with incentives, this acted as additional motivation to those who wanted to stop smoking: “It was win-win. I wanted to quit anyways so you had the benefit of not smoking and getting paid not to smoke”, and, “It was the icing on the cake. It was a nice perk. I had been thinking about it (quitting) for a long time and it gave me a slight push” [35]. In other words, incentives and opportunity impacted on perceived ability to quit or likelihood of quitting. However, for those smokers without any such priority, and with little perceived ability to quit, incentives made no difference: “It’s not about the money. It’s about the satisfaction of what I get from a cigarette”, and, “it’s a habit – an addiction. You can’t just be paid to work at it. You have to want it for yourself, not cause you’re getting paid” [35]. In other words, the priority an individual placed on quitting, and their perceived ability to do so, impacted on the likely effectiveness of any intervention and its components. Thus, although the data were not that “rich” or “thick” [42], it was still possible to articulate the relationships between concepts, and thus to create the new conceptual model.

The implications inherent in these findings were then considered. In this case, a working hypothesis might be that the priority an employee places on quitting, and their perceived ability do so, will moderate the effectiveness of any relevant workplace intervention. The relative importance given to these specific concepts was determined by the evidence: These were the only concepts that the evidence suggested were definite pre-requisites for successful quitting.

## Discussion

Evidence was found to support all of the key concepts in the *a priori* framework, i.e. beliefs about smoking, the pros and cons and perceived norms regarding smoking in the workplace, and the factors moderating the relationship between any intervention and successful quitting: Dependence, priority of quitting and self-efficacy (perceived ability to quit). The new concepts produced by the synthesis, perhaps unsurprisingly, related to the contextual specifics of the setting (the workplace) and the interventions (components, delivery etc.), which were not well represented in the behavioural theories that formed the basis of the *a priori* framework. The synthesis does not appear to be sensitive to the data collection methods employed by the included studies because all of the concepts in the model were informed by evidence from all included study designs. In the same way, the overall synthesis was not greatly affected by the type of intervention being examined in the included studies, though the concepts of employer support and employee attitudes to smoking had some distinct elements derived exclusively from studies exploring experiences of workplace bans and restrictions.

There is nothing to suggest that the workplace itself offers a particularly special setting for smoking cessation or reduction interventions, as the intervention preferences described and the key concepts of priority and perceived ability to quit can all exist outside the workplace. After all, unassisted smoking cessation is the most frequently successful approach [43]. However, the workplace does provide a context for interventions and this evidence synthesis suggests that employees’ expectations regarding employers’ support for, and enforcement of interventions or restrictions might facilitate smoking cessation. If an intervention is to take place in the workplace, then any benefits of that environment and context must be exploited.

The findings of this evidence synthesis suggest that the following elements of the *a priori* framework’s foundation theories represent the single most important concepts in smoking cessation theory within the workplace context: Motivation to change [17] and preparation for change [19], from versions of the stages of change model [9]; and Conrad’s *cue-to-action* addition to the Health Belief Model [21]. These ideas are captured by the broad concept of *readiness for change* in this paper’s conceptual model and, in particular, in the concepts of priority of quitting and perceived ability to quit. The concept of readiness for change is well-known in theories relating to smoking cessation, but this is the first evidence synthesis to validate that finding with specific reference to the evidence of employees’ views relating to workplace smoking programmes.

The findings of this synthesis are contextual and should be viewed alongside the effectiveness evidence. Indeed, they might help to explain differences in the apparent relative efficacy of interventions. Self-help methods, social and environmental support, and incentives and competitions, were all dismissed as ineffective by a Cochrane review [1], although some of the included trials reported that the priority of quitting variable might either predict cessation rates [44] or predict participation, and participation has been found to correlate with cessation [45,46]. However, this evidence synthesis suggests that such intervention components are liked by employees, particularly those for whom quitting is a priority and a perceived possibility. Indeed, competitions and incentives are thought to offer a valuable, additional motivation. The trials included in the Cochrane review that evaluated these interventions were aimed at all employees who smoke, regardless of readiness for change (for example, [47-49]), yet, as noted by this qualitative evidence synthesis, such incentives would be unlikely to have any effect unless the smoker was ready to quit. Interventions that have appeared most effective in terms of overall rates of cessation or reduction, such as group therapy and individual counselling [1], address the issue of health behaviour change more broadly, rather than through a single component, such as an incentive. They focus on altering employed smokers’ beliefs about smoking, to affect a change in opinions about the importance of, or need and ability to stop smoking, as well as seeking to motivate and sustain change. This evidence synthesis suggests therefore that there might be value in testing a multi-faceted intervention that includes components to appeal to all employed smokers, whatever stage they occupy in the process of change. An intervention should involve visible employer support, incentives, competitions, and self-help methods, as well as NRT, and individual and group counselling. Such an intervention would obviously be resource intensive, and its cost-effectiveness would need to be assessed, but it has the benefit of potentially addressing the needs and preferences of all employees who smoke.

### Strengths and limitations of the review

This review potentially possesses greatest external validity for north America, as this was the location for the majority of studies (USA), but there was great variety in terms of the sectors, age and gender groups covered; the different intervention types; and the range of organisations. However, the heterogeneity of organisations and sectors within the included studies prevented any evaluation of some variables within the synthesis, for example, whether intervention needs and preferences differ between socio-economic groups. Nevertheless, the body of evidence appears to be good quality, according to the reporting and dissonance criteria applied. The synthesis only included 15 studies, but the identification of evidence to support all 11 concepts in the *a priori* framework, and the absence of any substantial effect from the potential exclusion of inadequately reported studies, suggests that data saturation might have been reached.

## Conclusions

Employees’ views about smoking in the workplace, and their employers’ roles and responsibilities, are mostly shaped by their beliefs about smoking in general. Workplace interventions should have multiple components to satisfy the requirements of all employees who smoke, both those who feel ready and able to quit, and those for whom quitting is not yet a priority or a possibility. This is because the needs and preferences of these two groups can be quite different.

## Competing interests

The authors declare that they have no competing interests.

## Author contributions

CC conceived and designed the case study; CC, JR and JL extracted the data, appraised included studies and analysed and interpreted the data. CC drafted the paper, JR, JL, DF and AB undertook critical revision of important content of the manuscript. All authors approved the final version of the manuscript.

## Pre-publication history

The pre-publication history for this paper can be accessed here:

http://www.biomedcentral.com/1471-2458/13/1095/prepub

## Supplementary Material

Additional file 1 Example primary research studies search. Click here for file
