# Supplementary material for: A qualitative evidence synthesis of employees’ views of workplace smoking reduction or cessation interventions
Source: BMC Public Health. 2013 Nov 26;13:1095. doi: 10.1186/1471-2458-13-1095 (PMC4222886; doi:10.1186/1471-2458-13-1095)
Supplement: Additional file 1 — Example primary research studies search. [file 1471-2458-13-1095-S1.docx]

**Additional file 1: Example primary research studies search**

Database: PsycINFO <1987 to October Week 4 2011>

Search Strategy:

--------------------------------------------------------------------------------

1 exp Smoking Cessation/ (7104)

2 ((smoking or tobacco) adj2 (cess$ or quit$ or prevent$ or promot$) adj6 ((work$ or employ$ or organ$) adj3 (intervention$ or incentive$ or promotion$ or program$ or support$))).tw. (106)

3 1 or 2 (7122)

4 (workplace adj4 ((health or lifestyle) adj3 promotion)).tw. (169)

5 exp Qualitative Research/ (2907)

6 (questionnaire$ or survey$ or interview$ or focus group$ or view$ or experienc$ or opinion$ or attitude$ or perce$ or prefer$ or qualitative).tw. (916483)

7 5 or 6 (916579)

8 3 and 4 and 7 (0)

9 workplace.tw. (16101)

10 1 and 9 (100)

11 2 or 4 (274)

12 10 or 11 (354)

13 7 and 12 (186)
